# Mobile bearing shows larger rollback motion than fixed bearing in total knee arthroplasty using a medial stabilising technique with a navigation system

**DOI:** 10.1002/jeo2.12053

**Published:** 2024-06-12

**Authors:** Takahiro Tsushima, Eiji Sasaki, Shizuka Sasaki, Kazuki Oishi, Yukiko Sakamoto, Yuka Kimura, Hironori Otsuka, Yuji Yamamoto, Eiichi Tsuda, Yasuyuki Ishibashi

**Affiliations:** ^1^ Department of Orthopaedic Surgery Hirosaki University Graduate School of Medicine Hirosaki Japan; ^2^ Department of Orthopaedic Surgery Japan Community Health Care Organization Akita Hospital Noshiro Japan; ^3^ Department of Rehabilitation Medicine Hirosaki University Graduate School of Medicine Hirosaki Japan

**Keywords:** clinical outcomes, femoral rollback motion, intraoperative knee kinematics, medial stabilising technique, total knee arthroplasty

## Abstract

**Purpose:**

This study aimed to investigate the intraoperative knee kinematics of cruciate‐retaining total knee arthroplasty with a medial stabilising technique (MST‐TKA) and compare the kinematics between mobile‐ and fixed‐bearing MST‐TKAs. We hypothesised that mobile‐bearing MST‐TKA would result in greater physiological kinematic motion than fixed‐bearing MST‐TKA.

**Methods:**

Twenty‐one and 20 knees underwent mobile‐ and fixed‐bearing MST‐TKAs using a navigation system (Orthopilot® ver. 6.0; B. Braun Aesculap), respectively. In the preoperative and postoperative kinematic analysis, the knee was moved manually from 0° to 120°, and femoral anteroposterior translations of the medial femoral condyle (MFC) and lateral femoral condyle (LFC) were recorded every 0.1 s from 0° to 120°. Data were subsequently extracted from the software every 10° of flexion and compared between the two groups, and the correlation coefficients between preoperative and postoperative kinematics were calculated.

**Results:**

In the postoperative analysis, the MFC in the mobile‐bearing group showed significant posterior translation at 100°, 110° and 120° compared to the fixed‐bearing group (*p* < 0.01). Similarly, the LFC in the mobile‐bearing group showed significant posterior translation at 100°, 110° and 120° compared to the fixed‐bearing group (*p* < 0.05, *p* < 0.01 and *p* < 0.05, respectively). In the mobile‐bearing group, the preoperative and postoperative anteroposterior translations of the MFC and LFC were correlated (*p* < 0.01), while in the fixed‐bearing group, there was no correlation.

**Conclusion:**

The femoral rollback motion in the mobile‐bearing MST‐TKA correlated with the preoperative kinematics and was larger than that in the fixed‐bearing group.

**Level of Evidence:**

Level II, therapeutic prospective cohort study.

**Abbreviations**
3D: three dimensional
ADL: activities of daily living
AP: anteroposterior
CR: cruciate retaining
dMCL: deep medial collateral ligament
FTA: femoral tibial angle
HKAA: hip‐knee‐ankle angle
KOOS: Knee Injury and Osteoarthritis Outcome Score
LFC: lateral femoral condyle
MFC: medial femoral condyle
MST: medial stabilising technique
OA: osteoarthritis
ROM: range of motion
TKA: total knee arthroplasty
WBLR: weight‐bearing line ratio

## INTRODUCTION

For excellent outcomes after total knee arthroplasty (TKA), the physiological motion pattern is important [35]. However, previous studies have demonstrated nonphysiological kinematics post‐TKA, such as lateral pivot [1, 3] and parallel [1, 2] and paradoxical motions [36]. These movements are associated with diminished stability, abnormal sensations, decreased functional outcomes, restricted range of motion (ROM) and heightened challenges in performing activities of daily living (ADL). Accordingly, it is reasonable to assume that the nonphysiological kinematics are the primary contributor to the dissatisfaction experienced by nearly 20% of patients who underwent TKA [33, 34].

Recently, TKA with a medial stabilising technique (MST‐TKA) has been reported [19]. The MST‐TKA aims to minimise bone resection and soft tissue release from the medial aspect of the knee [13, 19, 27]. An intraoperative evaluation of MST‐TKA revealed that increased medial laxity was achieved with deep medial collateral ligament (dMCL) release and osteophyte resection [27]. Moreover, this approach has shown potential for achieving greater postoperative ROM compared to the conventional gap‐balancing technique [13]. However, the effects of MST‐TKA on postoperative knee kinematics remain unclear.

This study aimed to reveal the intraoperative knee kinematics after cruciate‐retaining (CR) MST‐TKA using a navigation system. The kinematic data of mobile‐ and fixed‐bearing MST‐TKAs were compared in terms of femoral rollback and rotation. Furthermore, the relationship between kinematics and clinical outcomes was investigated. We hypothesised that compared with fixed‐bearing MST‐TKA, mobile‐bearing MST‐TKA would result in greater physiological kinematic motion and better clinical outcomes. This is due to the main advantage of mobile‐bearing TKA, which allows for better function by facilitating femorotibial rotation and reducing contact stress [29].

## MATERIALS AND METHODS

### Subjects

This study was conducted from September 2020 to July 2022. Fifty‐two patients who underwent TKA for knee OA were screened. Eleven cases were excluded because of valgus alignment deformity, rheumatoid arthritis, post‐traumatic knee osteoarthritis (OA), severe bony defects requiring bone graft or metal augmentation, posterior‐stabilised TKA and revision TKA; for patients with a valgus knee, rheumatoid arthritis and post‐traumatic knee OA, such as severe bony defects requiring bone graft or metal augmentation, other prostheses were used. Finally, 41 patients who underwent primary CR MST‐TKA for varus knee OA were included in this study. Twenty‐one knees were performed using mobile‐bearing MST‐TKA (e.motion®; B. Braun Aesculap), referred to as the mobile‐bearing group, and 20 knees were performed using fixed‐bearing MST‐TKA (Columbus®; B. Braun Aesculap), referred to as the fixed‐bearing group. The TKA prothesis was selected at random by the surgeon.

### The prosthesis

In the mobile‐bearing group, the e.motion® enhances congruence and distributes constraint more evenly, reducing both wear on the polyethylene and the risk of tibial component loosening. Furthermore, the trochlear design, with its large‐radius groove, optimises leverage in the extension aid and ensures smooth patellar tracking, whilst reducing constraint and the risk of anterior knee pain. The polyethylene insert is allowed to move multidirectionally on top of the tibial tray, while a hook‐shaped peg on the tray prevents bearing dislocation. The Columbus® has an increased anterior lip and a more conforming articular surface for the prevention of anterior displacement of the condyles during knee flexion [10, 18]. Both femoral components have two radii, ensuring that the distal radius stays constant over a distance of 90° and has a large contact area with the tibial insert to localise stress peaks and transverse force.

### Surgical procedure

All TKAs were performed using a tourniquet and MST [13, 19, 27] using a computed tomography‐free navigation system (Orthopilot® ver. 6.0; B. Braun Aesculap). The measurement accuracy for calculating coronal and sagittal alignments in this navigation system was within ±1°. The detailed approach and procedure for MST has been previously described [13, 27]. Generally, although the medial compartment is tight, a lateral laxity of up to 3° is allowed. In addition, increased laxity is allowed in flexion than in extension. The medial release was confined to the dMCL. The dMCL was released by completely separating the tibial insertion of the meniscotibial ligament from the anterior to the posterior. Tibial resection was performed by cutting the bone perpendicular to the mechanical axis in the coronal plane. The tibia cutting block was positioned on the proximal tibia with a varus/valgus and anterior/posterior slope of 0° [13]. The required amount of resection was estimated based on the thinnest polyethylene insert. The amount of femoral bone resection required was determined based on the femoral component size. The distal femoral cutting angle was used to obtain a neutral mechanical alignment. The femoral cutting angle (femoral component rotational angle) was adjusted such that the flexion gap was 1–2 mm tighter than that on the lateral side, and internal rotation was avoided in all cases.

### Kinematics analysis using the navigation system

Intraoperative three‐dimensional (3D) kinematics were analysed using the navigation system. The 3D kinematic analyses were conducted preoperatively and postoperatively after the closure of the skin incision. Knee kinematics were assessed for nonweight‐bearing passive flexion in the supine position, with the heel supported and rotation stress withheld. The surgeon slowly flexed the hip and knee to the final point while allowing gravity to assist in knee flexion. In particular, the hip was allowed to move vertically to the ground, as abduction and adduction of the hip would change the rotation. During the measurement, the knee was taken through an ROM from maximum extension to maximum flexion. For the intraoperative kinematic analysis, the femoral anteroposterior (AP) translation of the medial femoral condyle (MFC) and lateral femoral condyle (LFC) and femoral rotation angle were recorded every 0.1 s in the software from 0° to 120° of flexion using the navigation system. These data were subsequently extracted from the software every 10° of flexion and analysed. Femoral rotation expressed the difference in rotation between the femoral and tibial trackers. In femoral AP translations, positive values showed posterior translation, whereas negative values showed anterior translation. In femoral rotations, positive values indicated external translation, whereas negative values indicated internal translation.

### Radiographic evaluation

Preoperative and 1‐year postoperative radiographic parameters were also measured. The mechanical axis was defined as the line from the centre of the femoral head to the centre of the talus on a weight‐bearing radiograph. A transverse line was drawn between the medial and lateral edges of the tibial plateau. The distance from the intersection of the transverse line and the mechanical axis to the medial edge of the tibial plateau was also measured. This distance was divided by the transverse diameter of the tibial plateau and defined as the weight‐bearing line ratio (WBLR). The hip‐knee‐ankle angle (HKAA) formed by the union of the mechanical axes of the femur and tibia was defined as an angular deviation from 180°. Varus deviations were defined as negative, and valgus deviations were defined as positive and measured on weight‐bearing radiographs. The femoral tibial angle (FTA) was measured using the AP view of the knee on weight‐bearing radiographs.

### Clinical evaluation

Preoperative and postoperative final follow‐up knee joint extension and flexion angles were recorded. In addition, as patient‐reported outcome measures, the Knee Injury and Osteoarthritis Outcome Score (KOOS) [26] was evaluated at the final follow‐up. The KOOS is attracting attention as a patient‐reported outcome measure. Conventional objective scales, such as the Japanese Orthopaedic Association and Knee Society Score (KSS) scores, mainly reflect the KOOS ADL scores in patients with postoperative TKA. Furthermore, there was a strong correlation between KOOS pain and KSS. Therefore, the KOOS is a useful tool for evaluating conditions after TKA [26].

### Statistical analyses

Statistical data are presented as medians [interquartile ranges]. All statistical data were evaluated for normality using the Shapiro–Wilk test. The Mann–Whitney *U* test was used to compare the demographic data, preoperative ROM and radiographic measurements, such as FTA, HKAA and WBLR between the mobile‐ and fixed‐bearing groups. In the kinematic analytical process, kinematic data at 10° increments from 0° to 120° were analysed. The Mann–Whitney *U* test was used for the comparison of kinematic results between the mobile‐ and fixed‐bearing groups. A post hoc power analysis was performed for the kinematic measurements of the mobile‐ and fixed‐bearing group comparisons. The correlations between preoperative and postoperative knee kinematics were evaluated using Spearman's correlation coefficients. The Mann–Whitney *U* test was used to compare the postoperative ROM, postoperative radiographic measurements and each KOOS subscale score. Finally, the correlation between each KOOS subscale, knee flexion angle at final follow‐up and femoral translation/rotation at 120° of flexion were determined using Spearman's correlation coefficients. Analyses were performed for both groups. Flexion angles at the final follow‐up were similarly analysed for correlation. For the AP translation (mm), the standard deviation was 3.5. If the true difference between the experimental and control means was 2.9, we rejected the null hypothesis that the population means of the experimental and control groups were equal with a probability (power) of 0.844. The type I error probability associated with the test of the null hypothesis was 0.05. Statistical analyses were performed using SPSS (version 29.0; SPSS Inc.), and *p* < 0.05 was considered statistically significant.

## RESULTS

### Patient demographics

Ten men and 31 women were enroled in the study, and their mean age was 72.5 ± 4.8 years. Twenty‐one and 20 patients underwent mobile‐ and fixed‐bearing TKAs, respectively. There were no significant differences in age, follow‐up period and preoperative ROM of the knee between the mobile‐ and fixed‐bearing groups. Preoperative measurements, such as FTA, HKAA and WBLR, also showed no significant differences between the groups (Table 1).

**Table 1:** Preoperative demographic data in the mobile‐ and fixed‐bearing groups.

Characteristics	MB (*n* = 21)	FB (*n* = 20)	*p* Value
Age (years)	73.0 [70.0–75.0]	74.0 [69.8–79.0]	0.266
Sex (male/female)	5/16	5/15	
Height (cm)	150.5 [147.6–157.2]	154.9 [150.2–159.4]	0.279
Body weight (kg)	62.7 [53.7–69.9]	67.8 [61.2–76.5]	0.160
BMI (kg/m^2^)	27.0 [24.7–29.2]	29.2 [25.7–31.6]	0.261
Follow‐up periods (months)	24.0 [21.8–26.3]	24.0 [22.1–25.1]	0.215
Preflexion angle (°)	125.0 [120.0–130.0]	120.0 [107.5–130.0]	0.260
Pre‐extension angle (°)	−10.0 [−15.0 to −5.0]	−10.0 [−10 to −5.0]	0.178
Pre‐range of motion (°)	115.0 [105.0–120.0]	112.5 [95.0–121.3]	0.978
Pre‐FTA (°)	181.1 [178.3–183.5]	182.5 [179.5–185.0]	0.499
Pre‐HKAA (°)	9.0 [6.0–11.0]	13.0 [9.5–15.0]	0.071
Pre‐WBLR (%)	12.0 [0.0–23.0]	7.3 [−15.8 to 15.0]	0.310

*Note*: Data are presented as medians [interquartile ranges]. The *p*‐value represents the comparison between the MB and FB groups performed using the Mann–Whitney *U* test.

Abbreviations: BMI, body mass index; FB, fixed bearing; FTA, femorotibial angle; HKAA, hip‐knee‐ankle angle; MB, mobile bearing; WBLR, weight‐bearing line ratio.

### Femoral AP translation analysis

**Figure 1:**
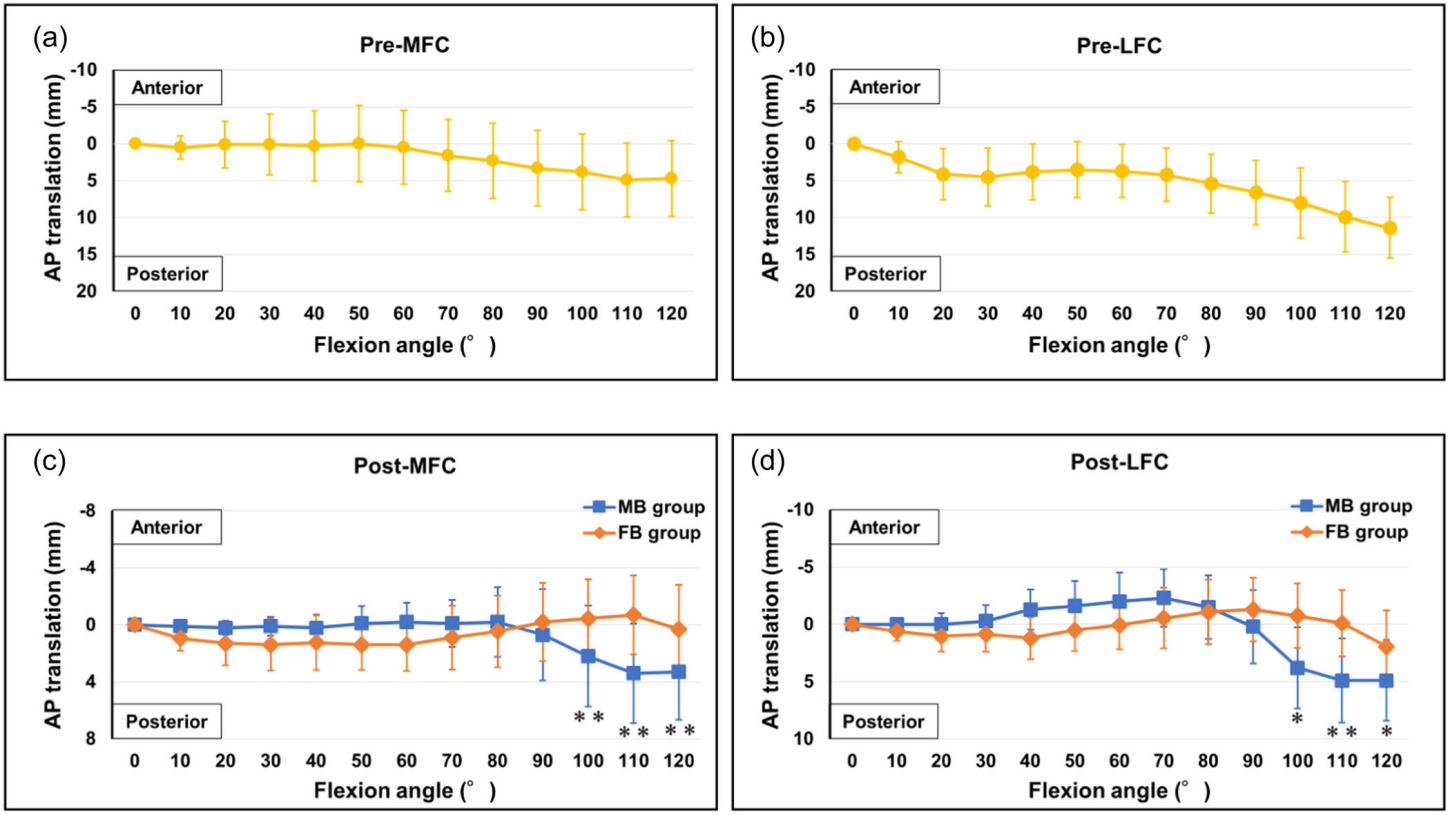
Preoperative and postoperative anteroposterior translations in the mobile‐ and fixed‐bearing groups. (a) Preoperative translations on the MFC. (b) Preoperative translations on the LFC. (c) Postoperative translations on the MFC. (d) Postoperative translations on the LFC. Comparisons between the MB and FB groups were performed using the Mann–Whitney *U* test (**p < 0.01; *p < 0.05). AP, anteroposterior; FB, fixed bearing; LFC, lateral femoral condyle; MB, mobile bearing; MFC, medial femoral condyle.

In the preoperative analysis, the MFC and LFC translated posteriorly in relation to the tibia from the initial period of flexion, showing a femoral rollback motion. The translations of the MFC and LFC were 4.7 [−0.5 to 8.0] mm (Figure 1a) and 11.4 [8.3–13.7] mm at 120° of flexion, respectively (Figure 1b).

In the postoperative analysis, the MFCs in the mobile‐bearing group showed significant posterior translation at 100°, 110° and 120° of flexion (2.2 [1.1–5.5], 3.4 [2.1–6.7] and 3.3 [2.0–6.6] mm, respectively) than those in the fixed‐bearing group (–0.5 [−2.9 to 2.1], –0.7 [−3.1 to 2.1] and 0.3 [−2.9 to 2.1] mm, *p* < 0.01, respectively; Figure 1c). Similarly, the LFCs in the mobile‐bearing group showed significant posterior translation at 100°, 110° and 120° of flexion (3.8 [1.0–6.7], 4.9 [2.1–8.3] and 4.9 [2.8–8.2] mm, respectively) than those in the fixed‐bearing group (–0.8 [−1.9 to 2.9], –0.1 [−1.1 to 2.9] and 2.0 [−0.3 to 3.9] mm and *p* < 0.05, *p* < 0.01 and *p* < 0.05, respectively; Figure 1d).

### Femoral rotation analysis

**Figure 2:**
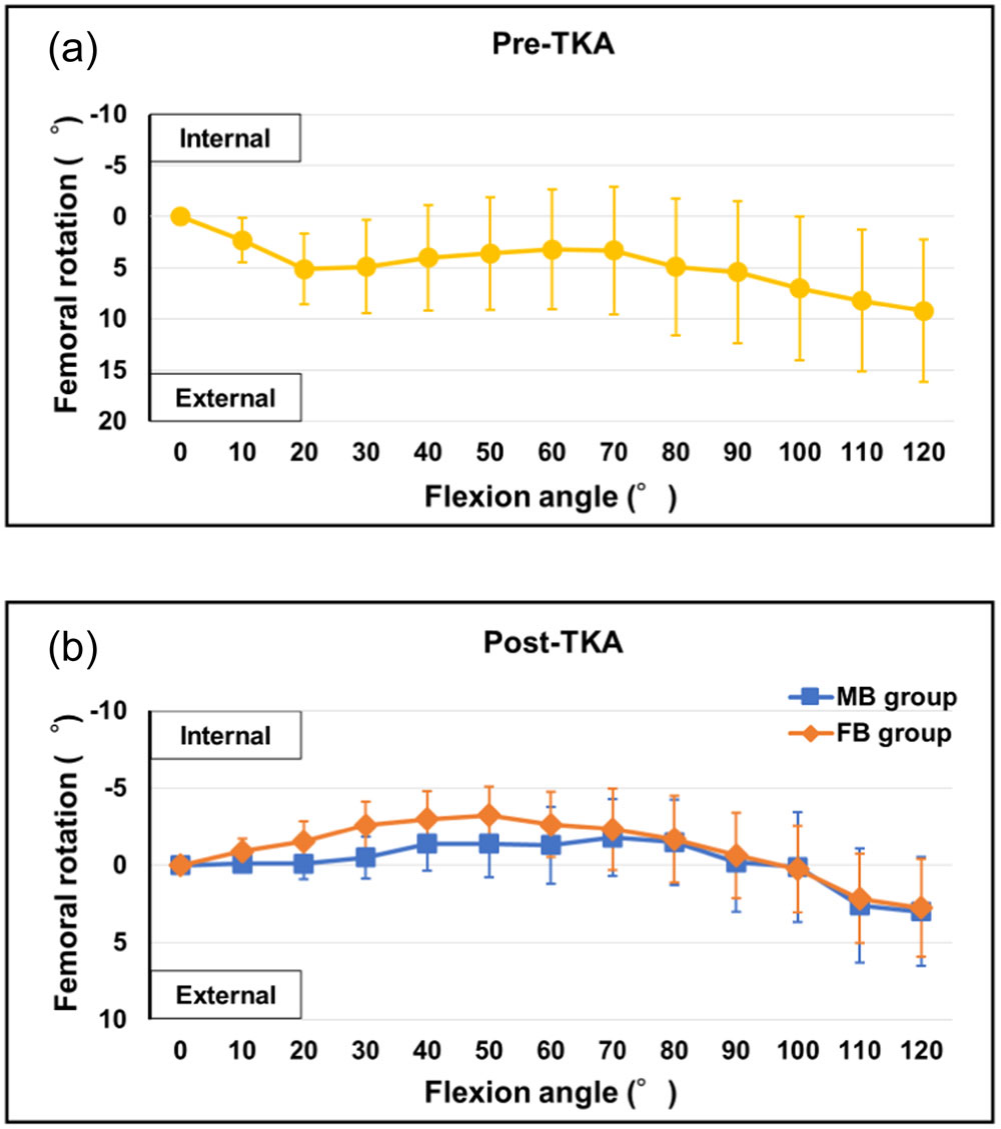
Preoperative and postoperative femoral rotation angles in the mobile‐ and fixed‐bearing groups. (a) Preoperative femoral rotation angles. (b) Postoperative femoral rotation angles. FB, fixed bearing; MB, mobile bearing; TKA, total knee arthroplasty.

Preoperatively, the femur rotated externally from 0° to 120° of flexion. The maximum external rotation angle was 9.2 [6.1–17.5]° at 120° of flexion (Figure 2a).

Postoperatively, the femurs in both groups rotated internally from 0° to a slight flexion position and then rotated externally to 120° of flexion. In the mobile‐bearing group, the femur rotated internally from 0° to 70° of flexion, after which it rotated externally. The maximum internal rotation angle was 1.8 [−0.6 to 3.2]° at 70° of flexion, and the maximum external rotation angle was 3.0 [0.0–3.7]° at 120° of flexion. In the fixed‐bearing group, the femur rotated internally from 0° to 50° of flexion, after which it rotated externally. The maximum internal rotation angle was 3.3 [−0.6 to 4.2]° at 50° of flexion, and the maximum external rotation angle was 2.8 [−1.6 to 4.7]° at 120° of flexion. There were no significant differences between the two groups (Figure 2b).

### Correlation coefficients between preoperative and postoperative knee kinematics

In the mobile‐bearing group, the preoperative and postoperative AP translations of the MFC (*r* = 0.246), LFC (*r* = 0.529) and femoral rotation angle (*r* = 0.308) were positively correlated (*p* < 0.001, respectively). In the fixed‐bearing group, only preoperative and postoperative femoral rotation was positively correlated (*r* = 0.423, *p* < 0.001) (Table 2).

**Table 2:** Correlation coefficients between preoperative and postoperative knee kinematics.

Characteristics	MB group		FB group	
	*r*	*p* Value	*r*	*p* Value
AP translation of MFC	0.246	<0.001**	–0.018	0.687
AP translation of LFC	0.529	<0.001**	0.046	0.302
Femoral rotation	0.308	<0.001**	0.423	<0.001**

*Note*: Values were represented using Spearman's correlation coefficients.

Abbreviations: AP, anteroposterior; FB, fixed bearing; LFC, lateral femoral condyle; MB, mobile bearing; MFC, medial femoral condyle.

** A *p*‐value < 0.01 was considered significant.

### Clinical outcomes and their correlation with knee kinematics

There was no significant difference in the postoperative ROM between both groups. Similarly, there were no significant differences in postoperative measurements, such as FTA, HKAA, WBLR and each KOOS score, between the groups (Table 3).

**Table 3:** Clinical outcomes and postoperative radiographic measurements in the mobile‐ and fixed‐bearing groups.

Characteristics	MB (*n* = 21)	FB (*n* = 20)	*p* Value
Postflexion angle (°)	120.0 [110.0–125.0]	115.0 [100.0–120.0]	0.248
Postextension angle (°)	0.0 [−5.0 to 0.0]	0.0 [−5.0 to 0.0]	0.340
Postrange of motion (°)	120.0 [105.0–125.0]	115.0 [100.0–120.0]	0.295
Post‐FTA (°)	176.5 [175.5–176.8]	176.0 [175.0–176.0]	0.052
Post‐HKAA (°)	0.0 [0.0–1.0]	1.0 [−1.0 to 1.0]	0.660
Post‐WBLR (%)	50.0 [49.0–50.0]	49.3 [47.6–50.3]	0.610
KOOS pain (points)	88.9 [86.1–92.4]	77.8 [77.0–88.9]	0.162
KOOS symptoms (points)	91.1 [85.7–96.4]	85.7 [75.0–90.0]	0.206
KOOS ADL (points)	86.8 [82.7–87.9]	70.7 [67.6–89.0]	0.139
KOOS sports/rec. (points)	55.0 [25.0–75.0]	50.0 [35.0–56.3]	0.933
KOOS QOL (points)	75.0 [57.8–81.3]	62.5 [56.3–87.5]	0.739

*Note*: Data are presented as medians [interquartile ranges]. Comparisons between MB and FB were performed using the Mann–Whitney *U* test. A *p*‐value < 0.05 was considered significant.

Abbreviations: ADL, activities of daily living; FB, fixed bearing; FTA, femorotibial angle; HKAA, hip‐knee‐ankle angle; KOOS, Knee Injury and Osteoarthritis Outcome Score; MB, mobile bearing; QOL quality of life; WBLR, weight‐bearing line ratio.

The correlation among the posterior translation of the MFC/LFC, the femoral rotation angle at 120° flexion, the postoperative flexion angle and each KOOS score at the final follow‐up was also assessed. In this analysis, the posterior translation of the MFC was positively correlated with the KOOS symptoms (*r* = 0.375, *p* = 0.027) and KOOS ADL (*r* = 0.341, *p* = 0.039). In addition, the posterior translation of the LFC was significantly correlated with the KOOS ADL (*r* = 0.375, *p* = 0.036; Table 4).

**Table 4:** Correlation between postimplantation knee kinematics and postoperative outcome scales.

	Posterior translation of MFC at 120°		Posterior translation of LFC at 120°		Femoral rotation angle at 120°	
	*r*	*p* value	*r*	*p* value	*r*	*p* value
Postflexion angle	0.100	0.533	0.023	0.885	−0.138	0.390
KOOS pain	0.294	0.078	0.179	0.288	−0.160	0.344
KOOS symptoms	0.375	0.027*	0.213	0.206	0.022	0.897
KOOS ADL	0.341	0.039*	0.356	0.036*	−0.099	0.577
KOOS sports/Rec.	0.028	0.867	0.019	0.913	−0.050	0.770
KOOS QOL	0.182	0.280	0.160	0.343	−0.053	0.755

*Note*: Values were represented using Spearman's correlation coefficients.

Abbreviations: ADL, activities of daily living; KOOS, Knee Injury and Osteoarthritis Outcome Score; LFC, lateral femoral condyle; MFC, medial femoral condyle; QOL quality of life.

* A *p*‐value < 0.05 was considered significant.

## DISCUSSION

The most important finding of this study was that the posterior translation, more commonly known as femoral rollback motion, of the mobile‐bearing group was larger than that of the fixed‐bearing group, correlating with the preoperative kinematics. Although the postoperative flexion angle and KOOS were not statistically different between the two groups, the posterior translation of the MFC was associated with the symptoms and ADL, and the posterior translation of the LFC was related to the ADL.

In the kinematics of knee extension, from 30° of flexion to full extension, the femur is internally rotated relative to the tibia, causing tightening of the cruciate ligament and locking of the joint, a phenomenon known as screw‐home movement [4]. In contrast, the kinematics of normal knee flexion show medial pivot motion, femoral external rotation with movement from full extension to 120° flexion and bicondylar rollback motion from 120° to maximum flexion [5, 16, 31, 32]. However, the kinematics after TKA exhibit large variations, including the medial pivot [23], lateral pivot [1, 3], parallel motion patterns [1, 2] and paradoxical motion [36].

There have been several reports regarding intraoperative kinematics during TKA [6, 7, 8, 9, 11, 12, 14, 15, 17, 20, 21, 22, 24, 25, 28, 30, 35] using a navigation system. Kinoshita et al. [15] reported that the normal rotational kinematics of the knee can be restored by controlling soft‐tissue balance during surgery and that medial rotational stability is important for the ROM responsible for inducing the ideal rotational movement. Additionally, Matsumoto et al. reported that semimembranosus release reduces tibial internal rotation and flexion angle. Seon et al. [28]. evaluated the preoperative and postoperative kinematics following CR TKA. Their findings revealed a significant positive correlation between the degree of posterior rollback and external femoral rotation measured after TKA and those observed preoperatively.

In this study, postoperative femoral AP translation and rotation were significantly positively correlated with the preoperative kinematics in mobile‐bearing CR TKA. However, in the fixed‐bearing group, a correlation between preoperative and postoperative kinematics was only observed for rotation. Some in vivo studies have suggested that the kinematics of mobile‐bearing implants reproduce the internal rotation of the tibia more closely during flexion than fixed‐bearing implants [29, 36]. Thus, the relative movement of polyethylene on the tibial tray was thought to have caused femoral rollback in the mobile‐bearing group.

It is concerning that nearly 20% of patients, particularly younger patients, are not fully satisfied after TKA [33]. Many patients complain of decreased knee joint stability, a feeling of abnormality in the knee, poor functional outcomes, decreased ROM and difficulty performing daily activities [34]. These symptoms most likely arise due to the effects of nonphysiological kinematic motions. In particular, paradoxical motion is more pronounced in the early stages of knee flexion in CR TKA, when weight pushes the femur forward along the tibia until it is stopped by the posterior cruciate ligament, termed midflexion instability [36].

In this study, there were no instances of paradoxical motion in the early flexion phase for both mobile‐bearing and fixed‐bearing MST‐TKAs. This suggests that midflexion instability may not have occurred following MST‐TKA. With the MST‐TKA, medial soft tissue release and bone resection are minimised, and the degree of lateral laxity is acceptable. Therefore, the soft tissue balance is considered to be similar to that of a normal knee. The placement of a prosthesis that fits the appropriate soft tissue balance would have a positive impact on postoperative kinematics, leading to a reduction in postoperative pain, improvement in symptoms and even improvement in ADL and quality of life. In the present results, the rollback motion of MST‐TKA was also correlated with the symptoms and ADL of patient‐reported outcomes.

This study has some limitations. First, this study was not a randomised controlled trial. The sample size of each group was small, and the variation in the results among the patients was large. Therefore, it was difficult to perform a reasonable statistical analysis. A randomised controlled trial with a larger population is required to draw statistically validated conclusions. Second, the kinematic measurements were evaluated in nonphysiological conditions, such as under general anaesthesia, with use of a tourniquet, and in nonweight‐bearing conditions in a supine position. In addition, the lower limbs were moved manually in the kinematic measurement, which may cause unpredictable risks of knee rotation. In vivo kinematics should be evaluated using fluoroscopy or point clusters during postoperative activities under weight‐bearing conditions, such as walking. Third, the insert and component types were different as we used e‐motion® for the mobile‐bearing group and Columbus® for the fixed‐bearing group.

## CONCLUSION

The femoral rollback motion in the mobile‐bearing CR TKA correlated with the preoperative kinematics and was larger than that in the fixed‐bearing CR TKA. The posterior translation of the MFC correlated with symptoms and ADL, and the posterior translation of the LFC correlated with ADL.

## AUTHOR CONTRIBUTIONS

Takahiro Tsushima was responsible for the organisation and coordination of this study. All authors contributed to the management of this study and acquisition, analysis and interpretation of the data. All the authors have approved the manuscript for publication.

## CONFLICT OF INTEREST STATEMENT

The authors declare no conflict of interest.

## ETHICS STATEMENT

This study was approved by the ethical committee of Hirosaki University (2019‐596‐1). Informed consent was obtained from all study participants.

## Data Availability

The data that support the findings of this study are openly available.
